# Role of Decorin in Posterior Capsule Opacification and Eye Lens Development

**DOI:** 10.3390/cells10040863

**Published:** 2021-04-09

**Authors:** Shinsuke Shibata, Naoko Shibata, Satoshi Ohtsuka, Yasuo Yoshitomi, Etsuko Kiyokawa, Hideto Yonekura, Dhirendra P. Singh, Hiroshi Sasaki, Eri Kubo

**Affiliations:** 1 Department of Ophthalmology, Kanazawa Medical University, Ishikawa 9200293, Japan; shinsuke.shiba@gmail.com (S.S.); n-shiba@kanazawa-med.ac.jp (N.S.); mogu@kanazawa-med.ac.jp (H.S.); 2 Medical Research Institute, Kanazawa Medical University, Ishikawa 9200293, Japan; sohtsuka@koto.kpu-m.ac.jp; 3 Laboratory for Experimental Animals, Kyoto Prefectural University of Medicine, Kyoto 6028566, Japan; 4 Department of Biochemistry, Kanazawa Medical University, Ishikawa 9200293, Japan; yositomi@kanazawa-med.ac.jp (Y.Y.); yonekura@kanazawa-med.ac.jp (H.Y.); 5 Department of Oncogenic Pathology, Kanazawa Medical University, Ishikawa 9200293, Japan; kiyokawa@kanazawa-med.ac.jp; 6 Department of Ophthalmology, University of Nebraska Medical Center, Omaha, NE 68198, USA; dpsingh@unmc.edu

**Keywords:** Decorin, posterior capsule opacification, epithelial-mesenchymal transition, lens development, wound healing

## Abstract

Decorin (DCN) is involved in a variety of physiological and pathological processes. Epithelial-mesenchymal transition (EMT) of lens epithelial cells (LECs) has been proposed as a major cause for the development of posterior capsule opacification (PCO) after cataract surgery. We investigated the plausible target gene(s) that suppress PCO. The expression of *Dcn* was significantly upregulated in rat PCO tissues compared to that observed in the control using a microarray-based approach. LECs treated with fibroblast growth factor (FGF) 2 displayed an enhanced level of *DCN* expression, while LECs treated with transforming growth factor (TGF)β-2 showed a decrease in DCN expression. The expression of tropomyosin 1 (*Tpm1*), a marker of lens EMT increased after the addition of TGFβ-2 in human LEC; however, upregulation of *Tpm1* mRNA or protein expression was reduced in human LECs overexpressing human DCN (hDCN). No phenotypic changes were observed in the lenses of 8- and 48-week-old transgenic mice for lens-specific hDCN (*hDCN-Tg*). Injury-induced EMT of the mouse lens, and the expression patterns of α smooth muscle actin, were attenuated in *hDCN-Tg* mice lenses. Overexpression of DCN inhibited the TGFβ-2-induced upregulation of Tpm1 and EMT observed during wound healing of the lens, but it did not affect mouse lens morphology until 48 weeks of age. Our findings demonstrate that DCN plays a significant role in regulating EMT formation of LECs and PCO, and suggest that for therapeutic intervention, maintenance of physiological expression of DCN is essential to attenuate EMT progression and PCO formation.

## 1. Introduction

According to the latest report of the World Health Organization (WHO), cataract is the primary cause of blindness in the world, which affected 20 million people in 2010 (WHO. Global data on visual impairments 2010. WHO/NMH/PBD/12.01). Although cataracts can be cured surgically, the process may be difficult to perform even in many developing countries [1]. In addition, after cataract surgery, aberrant cell proliferation, fibrosis, and lens regeneration under the lens capsule lead to fibroblastic opacification and secondary visual impairment, known as the posterior capsule opacification (PCO) or after-cataracts. Currently, Nd-YAG laser posterior capsulotomy is the only treatment for PCO. However, after this laser treatment, postoperative uveitis, cystoid macular edema, transient increase in intraocular pressure, retinal detachment, and intraocular lens damage may occur as complications. In addition, Nd-YAG laser capsulotomy is occasionally unavailable in developing countries, leading to surgery-associated blindness. Furthermore, early cataract surgery and refractive correction are necessary to prevent amblyopia in cases of pediatric cataracts [2]. However, visually significant PCO hinders visual rehabilitation, increasing the probability of deprivation amblyopia after pediatric cataract surgery. PCO develops in 100% of eyes under 4 years of age if the posterior capsule of the lens remains intact [3]. Thus, secondary surgery for capsulotomy is occasionally required in younger children using general anesthesia [3]. Furthermore, epithelial-mesenchymal transition (EMT) and PCO should be regulated during re-growth of lens epithelial cells (LECs) for clinical treatments using accommodative lens refilling [4] and for the regeneration of clear crystalline lens in human eyes after cataract surgery.

Transforming growth factor (TGF) β signaling plays a significant role in the pathobiology of human anterior subcapsular cataract [5,6,7,8] and PCO [1,7,9,10]. In addition, TGFβ induces tissue fibrosis, EMT, and apoptosis [11,12,13,14] by upregulating genes encoding α-smooth muscle actin (αSMA), collagen types I and III, and tropomyosin (Tpm). Previous studies have revealed that fibroblast growth factor (FGF) is also involved in the stimulation of lens fiber differentiation [15,16] and mitosis, increasing the formation of collagen in PCO [17]. In our previous study, we observed that Tpm 1 and 2 are involved in regulating and stabilizing actin microfilaments and are induced by TGFβ-2 during EMT in LECs. Importantly, we found that FGF2 acts as an antagonist of TGFβ-mediated EMT progression [12]. Furthermore, we demonstrated that the expression of rat tropomyosin and human TPM1 and TPM2 was highly elevated in a rodent model of PCO and human cataractous lens capsules with LECs, suggesting their association with EMT and lens regeneration during PCO progression [18]. However, the molecular mechanisms underlying the progression and inhibition of PCO have not yet been clarified.

In this study, we aimed to analyze the changes in gene expression patterns during rat and mouse PCO formation in vivo using a microarray-based approach and real-time quantitative polymerase chain reaction (RT-qPCR). Furthermore, we analyzed the role of Decorin (*DCN*), the level of which increased markedly in rat PCO tissues, and during EMT and lens development. To achieve the impact of *DCN* expression on eye lens in vivo, we generated lens-specific human *DCN* transgenic mouse (*hDCN-Tg*). In addition, to establish a correlation between results obtained in vitro and *hDCN-Tg* in vivo, we assessed the levels of human DCN protein and mRNA in human aqueous humor and LECs of cataractous patients. We first found that abundant expression of *Dcn* is involved in EMT and that may result in PCO formation. Thus, the results of the study increase our understanding of the role of *Dcn* in EMT and PCO formation and plausible the related molecular mechanism underlying its effects.

## 2. Materials and Methods

### 2.1. Animals

All animal experiments were approved by the Kanazawa Medical University Ethics Committee for Animal Experiments (authorization No. 2018–3) and were conducted in accordance with the National Institutes of Health Guide for the Care and Use of Laboratory Animals and the Institutional Guidelines for Laboratory Animals from the Kanazawa Medical University. Sprague-Dawley (SD) rats, and C57BL/6J, C57BL/6JJmsSlc, and Slc:ICR mice were purchased from Sankyo Laboratories Japan (Ishikawa, Japan).

### 2.2. Surgical Procedure for Generation of PCO Animal Model

Method for extracapsular clear lens extraction (ECLE) from rat and mouse eyes was established, confirming the histological observation of rat PCO in our previous study [19]. Eighteen 7-week-old female albino SD rats and twelve 7-week-old C57BL/6J mice were used as PCO animal models. ECLE was performed in both eyes of all rats and mice anesthetized with intraperitoneal administration of a combination anesthetic prepared with 0.3 mg/kg body weight medetomidine, 4.0 mg/kg body weight midazolam, and 5.0 mg/kg body weight butorphanol (Wako, Osaka, Japan). Animals were sacrificed at either 0 (after the surgery was completed) (day 0), 7 (1 week (W)), or 14 days (2W) after surgery by administering a lethal dose of CO_2_. Mice exhibited no signs of distress during euthanasia. Euthanasia by CO_2_ inhalation was performed according to the American Veterinary Medical Association (AVMA) Guidelines for the Euthanasia of Animals. Lens capsules with LECs removed from all eyes were used as PCO samples. All PCO samples from right eyes were processed for microarray studies (*n* = 6 at each time point) and all PCO samples from left eyes were used for reverse transcription-quantitative PCR (RT-qPCR) (*n* = 4 at each time point) and protein blotting (*n* = 2 at each time point).

### 2.3. RNA Extraction

Total RNAs from rat and mouse PCO, human LEC samples, cultured mouse LECs (MLEC), and Simian virus 40-transformed human LECs (SRA01/04) (SRA-HLECs) were extracted using the RNeasy micro kit (Qiagen Inc., Valencia, CA, USA) following the manufacturer’s instructions. Samples of RNA were set aside for RT-qPCR to verify the results obtained from the microarray analysis. Total RNA quality was assessed by determining UV 260/280 absorbance ratios and examining RNA size distribution on NanoDrop ND-1000 (NanoDrop Technologies, Inc., Wilmington, DE, USA) processed on the Agilent 2100 Bioanalyzer (Agilent Technologies, Santa Clara, CA, USA) using the total RNA electrophoresis program. The purity and integrity of RNA were examined and validated as described previously [20]. The quality of total RNA was analyzed by evaluating the RNA integrity number (RIN) using Bioanalyzer RNA analysis (Agilent Technologies Japan Ltd., Tokyo, Japan). All RNA samples showed RIN > 9.0.

### 2.4. Microarray and Gene Ontology Analysis

Three rat PCO samples from each group on day 0, 1 W, and 2 W were used for microarray analysis to screen genes associated with rat PCO. All samples were processed for microarray analysis as follows: RNA labeling and hybridization were performed using GeneChip^®^ 3′ IVT PLUS reagent kit (Affymetrix, Inc. Santa Clara, CA, USA) according to the manufacturer’s protocol. Labeled cDNA was hybridized to Affymetrix^®^ GeneChip^®^ rat genome 230 2.0 array (Affymetrix, Inc.) and scanned with a GeneChip^®^ Scanner 3000 7G (Affymetrix, Inc.). The scanned images were analyzed with AGCC (Affymetrix^®^ GeneChip^®^ Command Console^®^ Software (v.10.7.3.1)) and Affymetrix^®^ Expression ComsoleTM (Affymetrix, Inc.).

Per chip normalization was performed by dividing each gene’s value by the specific control values or by the average intensity in the single array. Normalized data were exported for subsequent analysis. Genes with normalized ratios more than 2.0-fold or less than 0.5-fold were selected as significant genes.

### 2.5. RT-qPCR Validation

RT-qPCR was used to validate the significantly affected genes selected using microarray analysis and to analyze *Dcn* expression in mouse PCO samples and cultured MLECs and HLECs. To assess the expression of human *DCN*, rat and mouse *Dcn*, rat *Tpm2*, mouse and human *Tpm1*, and TGF β-induced (*Tgfbi*) mRNAs, we conducted relative quantification of mRNA using Prism7300 (Applied Biosystems^®^, ThermoFisher Scientific Japan Ltd., Tokyo, Japan). Comparative Ct method was used for relative quantification of mRNA expression. PCR amplification was performed using a TaqMan universal master mix and pre-developed rat, mouse, and human *Dcn* (Hs00754870_s1, Cat# 4331182), rat *Tgfbi* (Rn01442102_m1, Cat# 4331182), rat *Tpm2* (Rn01439798_g1, Cat# 4351372), and mouse *Tpm1* (Hs00165966_m1, Cat# 4331182) and human *Tpm1* (Hs04398572_m1, Cat# 4331182) probe mix (Applied Biosystems^®^). The relative quantity of each mRNA was determined using the comparative Ct method and then normalized using a pre-developed TaqMan 18S ribosomal RNA (18s rRNA) VIC probe or glyceraldehyde-3-phosphate dehydrogenase (GAPDH) as an endogenous control (Applied Biosystems^®^).

### 2.6. Construction of Green Fluorescent Protein (GFP)-hDCN Vector and Lentivirus Production

For generating cells (tissues) secreting hDCN protein in vitro, we used a lentivirus expression system (SBI System Biosciences, Palo Alto, CA, USA). Briefly, *hDCN* was amplified by PCR using the following primers; forward primer 5′-tggatccgccgccaccatgaaggccactatcatcctc -3′ and reverse primer 5′-agaattcttacttatagtttccgagttgaatgg-3′. The fragment was sub-cloned into the pBRBlue vector and confirmed by sequencing. Then, the *hDCN* fragment was excised using BamHI and EcoRI and sub-cloned in the pCDH1-EF1-copGFP (SBI) vector (pCDH-CMV-*hDCN*-EF1-copGFP) (SBI). Lentivirus particles were then produced in HEK293TN cells (System Biosciences) following transfection of pCDH-CMV-*hDCN*-EF1-copGFP vector with packaging vectors, psPAX2 and pMD2.G (deposited by Didier Trono, Lausanne, Switzerland: Addgene plasmid #12260 and #12259 respectively, Addgene, MA). Lentivirus particles were collected from the supernatants of 48 h culture media and concentrated with PEG-it solution (System Biosciences). Lentivirus infectious units (IFU) were determined by the serial dilution method to adjust copy numbers for each experiment.

### 2.7. Cell Culture

Primary cultured MLECs were generated from 6-week-old Balb/C mice (*n* = 8) as described previously [21]. MLECs were maintained in Dulbecco’s modified Eagle’s media (DMEM; Wako) with 10% fetal bovine serum (FBS; Sigma, St. Louis, MO, USA) at 37 °C in a 19:1 air/CO_2_ atmosphere as described previously [21]. Cells from passages 5–7 were used for the experiments. Human LECs (SRA01/04: SRA-HLECs) were kindly gifted by Dr. Nobuhiro Ibaraki (Ibaraki Eye Clinic, Tochigi, Japan). SRA-HLECs were cultured in DMEM supplemented with 20% FBS (Sigma) at 37 °C in a 19:1 air/CO_2_ atmosphere.

To assess the effects of FGF2 (PeproTech, RockyHill, NJ, USA) or TGFβ-2 (R & D Systems Inc., Minneapolis, MN, USA), MLECs or SRA-HLECs were plated in triplicate in 35-mm culture dishes (TPP^®^ Techno Plastic Products AG, Trasadingen, Switzerland). Cells growing in DMEM containing 0.1% bovine serum albumin (BSA) (Wako) in the presence or absence of various test growth regulators received 0–100.0 ng/mL FGF2 or 0 to 10 ng/mL TGFβ-2 every other day for up to four days.

To determine the effect of DCN overexpression, the pCDH-CMV-*hDCN*-EF1-copGFP lentivirus was used to generate eukaryotic cells overexpressing GFP in the cytoplasm, and secreting *hDCN* into the medium. SRA-HLECs were cultured in DMEM supplemented with 20% FBS. For the negative control, the pCDH1-EF1-copGFP (SBI) lentivirus (GFP-Vec) was transduced into SRA-HLECs.

### 2.8. Western Blotting

Protein lysates of mouse PCO samples were prepared in ice-cold radioimmunoprecipitation assay (RIPA) buffer. Culture medium was collected and concentrated using Amicon^®^ Ultra-0.5 centrifugal filters (Merck Millipore Ltd. County Cork, Ireland) after transduction of GFP–hDCN and GFP-Vec to confirm the secretion of hDCN from SRA-HLECs. For western blotting, the concentrated medium or protein samples were treated with 0.2 U/mL protease-free chondroitinase (CSase)-ABC (C-ABC) (Sigma) in a solution of 0.01 M Tris, 0.01 M NaCl, and 0.012 M NaOAc containing 0.1% BSA for 1 h at 37 °C to digest the glycosaminoglycan chain, thereby exposing the core protein of DCN before sodium dodecyl sulfate-polyacrylamide gel electrophoresis (SDS-PAGE).

SDS-PAGE and western blotting were performed as described previously [22,23]. The membranes were probed with anti-rabbit DCN polyclonal antibody (Abcam Cat# ab137508, RRID:AB_2847890; Abcam, Cambridge, MA, USA) for human and mouse samples and anti-rabbit DCN polyclonal antibody (Cat# PA5-95830, RRID:AB_2807632, ThermoFisher Scientific Japan Ltd.) for rat samples. Anti-rabbit glyceraldehyde 3-phosphate dehydrogenase (GAPDH) polyclonal antibody (Cat# G9545, RRID:AB_796208; Sigma) was used to demonstrate equal protein loading in each lane.

### 2.9. Constructs for the hDCN-Tg Line

All animal and recombinant DNA experiments were approved by the Kanazawa Medical University Ethics Committee for Animal Experiments (authorization No. 2018-3) and Safety Committee for Recombinant DNA Experiments (authorization No. 2018-8). A plasmid in which Pax6-human αA-crystallin (P6a) composite promoter (CPV14) [24] drives *hDCN* cDNA was generated using the following procedures. Briefly, *hDCN* cDNA was amplified using the primers; 5′-tggatccgccgccaccatgaaggccactatcatcctc-3′ and 5′-agaattcttacttatagtttccgagttgaatgg-3′. The fragment was sub-cloned in the pBRBlue vector and confirmed via sequencing. Then, the *hDCN* fragment was digested using BamHI and EcoRI and sub-cloned in the pCPV14 plasmid. The P6a-*hDCN*-pA fragment, excised using NotI and XbaI, was cloned in the pBRPy-IRES-nLacZpA plasmid (the final product was pBRPy-P6a-hDCN-IRES-nLacZpA). This construct has the following two advantages: 1) *hDCN* expression was restricted within the crystalline lens using the P6a promoter in mice, which 2) can be readily confirmed using LacZ staining in tissues.

### 2.10. Generation of hDCN-Tg

The pBRPy-P6a-hDCNiLacZ vector was linearized with PmlI and purified using the QIAquick gel extraction kit (Qiagen Inc.). The fragment was used to generate transgenic mice by injecting into the pronucleus of fertilized eggs (C57BL/6JJmsSlc). Then, the eggs were transferred into the oviduct of pseudo-pregnant mice (Slc:ICR). Offspring were genotyped using the following primer set; 5′- tacagccatccaccttcagatgt -3′ and Rv 5′-gccacatatcctgatcttccaga-3′. The PCR product was 771 bp in length. Offspring harboring the exogenous fragment were bred with the C57BL/6JJmsSlc strain of mice.

### 2.11. Histochemical Staining for LacZ with X-Gal

Three *hDCN-Tg* and three wild-type (WT) mouse heads were collected from mice on postnatal day 2 (PD2) and fixed with 4% paraformaldehyde (PFA)/phosphate-buffered saline (PBS) (Nacalai Tesque Inc., Kyoto, Japan). The fixed tissue were then rinsed twice in PBS for 20 min × 2 times and placed in staining media containing 5 mM potassium ferricyanide/5 mM potassium ferrocyanide (Nacalai Tesque Inc.) 1/mg/mL 5-bromo-4-chloro-3-indolyl-β-D-galactoside (X-gal) (Nacalai Tesque Inc.)/0.02% NP40 (Sigma)/0.01% deoxycholate (Wako)/1 mM MgCl_2_ (Wako) in PBS. The tissues were incubated in staining media overnight in the dark at room temperature. After incubation, the tissues were washed twice with PBS and then post-fixed with 4% PFA/PBS for 2 h before paraffin embedding and sectioning. Hematoxylin and eosin (H & E) staining of the tissue sections was performed for contrast histological observation.

### 2.12. Microscopic, Histological, and Immunohistochemical Analyses

The eyes of 8 and 48-week-old *hDCN-Tg* and WT mice were fixed for 48 h in Super Fix KY-500 solution^®^ (Kurabo, Tokyo, Japan), embedded in paraffin, and sectioned at approximately 4 µm (*n* = 4). Then, the eyes were stained with H & E. For immunohistochemical analysis, the eyes were immunostained using a tyramide signal amplification (TSA^TM^) kit (Molecular Probes Inc., ThermoFisher Scientific Japan Ltd.), following the manufacturer’s protocol and as described previously [25,26]. For DCN immunostaining, the sections were treated with target retrieval solution (Dako/Agilent, Santa Clara, CA, USA) following treatment with 0.2 U/mL protease-free C-ABC as described in the section on western blotting to expose the core protein before blocking with a blocking reagent (Molecular Probes Inc., ThermoFisher Scientific Japan Ltd.). DCN was visualized using anti-rabbit DCN polyclonal antibody (ab137508; abcam). The cell nuclei were stained with 4′,6-diamidino-2-phenylindole (DAPI) (Fluoroshield Mounting Medium with DAPI: ImmunoBioScience Corp., Mukilteo, WA, USA). For negative controls (NC), rabbit and mouse IgG isotype controls (Dako) were used, and the primary antibody was omitted. NC was performed along with the experiments.

### 2.13. In Vivo Wound Healing Assay

The wound healing model was generated in 14-week-old mice as described previously [27]. Briefly, six *hDCN-T*g and six WT mice were anesthetized via isoflurane USP (Abbott Laboratories Japan, Tokyo, Japan) inhalation. Following topical application of mydriatics, the central anterior lens capsule was pierced once with the blade of a 27-gauge needle to a depth of approximately 2.5 mm from the corneal surface. After injection, the mice were allowed to heal for 5 and 10 days. The enucleated eye globes were fixed and embedded in paraffin for histological examinations after H & E staining. Immunohistochemical analysis using anti-αSMA Ab was performed as described in the previous section.

### 2.14. Obtaining LEC and Aqueous Humor Samples from Patients with Cataract

This study was approved by the ethics committee of the Kanazawa Medical University (Approval ID: R263). All patients at the Kanazawa Medical University, Japan, provided informed consent for participation. In addition, this study adhered to the tenets of the Declaration of Helsinki (2004) [28]. In total, 101 cataractous eyes of Japanese patients aged 19–87 years, who underwent cataract surgery at the Kanazawa Medical University between March and December 2015, were prospectively and sequentially examined. Patients with prior history of ocular surgery, trauma, active ocular disease (including retinitis pigmentosa, diabetes, retinal detachment, uveitis, vitreous hemorrhage, and/or receiving steroid medication) were excluded. The type and severity of the cataracts were graded and recorded based on a WHO classification system [29]. Three slit-lamp images were used for grading nuclear opalescence (N0-3), three retroillumination images for cortical cataracts (C0-3), and three retroillumination images for posterior subcapsular (P0-3) cataracts. Each scale on the WHO classification system is a devitalized grade ranging from 0 (clear or opacification less than grade1) to 3 (upper value on the N, C, and P scales).

### 2.15. Enzyme-Linked Immunosorbent Assay (ELISA) for DCN in the Aqueous Humor

The DCN levels in aqueous humor samples were measured using DCN ELISA kits (Sigma) according to the manufacturer’s instructions. Briefly, an aqueous humor sample or standard DCN recombinant protein was added to each well and incubated overnight at 4 °C. An anti-human DCN antibody was added following incubation with a streptavidin-horse radish peroxidase-conjugated antibody and substrate solutions. The absorbance at 450 nm of each well was recorded using an ELISA plate reader (Bio-Rad Laboratories, Hercules, CA, USA). Serial dilutions (0–700 pg/μL) of purified DCN protein were used as standards in each experiment. The detection limits of DCN in the aqueous humor ranged between 0.96 and 700 pg/mL. All experiments were repeated at least thrice, and the mean DCN levels (pg/mL) were calculated and presented.

### 2.16. Statistical Methods

For all quantitative data collected, the statistical analysis was conducted using one-way analysis of variance (ANOVA) test and Tukey’s test. The data are presented as mean  ±  SD of the indicated number of experiments. Significant differences between the control and treatment groups were defined at *p*-value < 0.05 for two or more independent experiments.

The correlation between cataract score, age, and DCN protein levels in human aqueous humor or *Dcn* mRNA levels in human LECs was analyzed using one-factor analysis of variance or Pearson’s correlation coefficient, with data expressed as mean ± standard error (SE). The power analysis to calculate the sample size of this study was performed. All statistical analyses and power analysis were performed using StatMate2.0 (GraphPad Software, Inc. La Jolla, CA, USA), and *p*-values less than 0.05 were considered statistically significant.

## 3. Results

### 3.1. Screening of Gene Expression Profile

Samples extracted from lens capsules at day 0 (control), and 1 and 2 W after ECLE from rats were used for microarray analysis. The data for the microarray analysis were deposited in the Gene Expression Omnibus (GEO) database (accession number: GSE152593).

**Figure 1:**
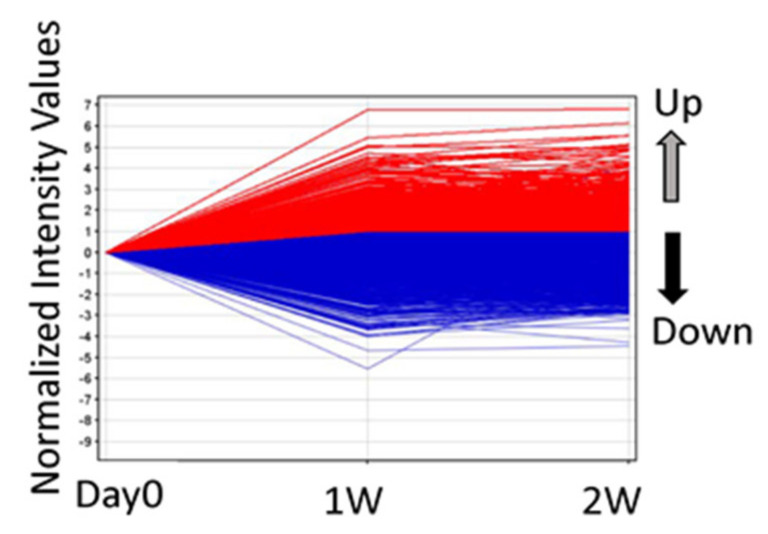
Differences in gene expression profile between 1 week (W) and 2 W groups after extracapsular clear-lens extraction (ECLE). Scatter plots were used to distinguish the differentially expressed mRNA. Red and blue indicate high and low expression, respectively.

First, among the approximately 28,700 well-substantiated rat genes on the array, 490 genes showed fold change > 2.0 1 W and 2 W after ECLE compared to that in the control (Figure 1).

Nine genes showed significant changes of less than 0.5-fold at 1 W and greater than 2.0-fold at 2 W after ECLE compared to that in the control (Figure 1). Compared to that in the control, 504 genes showed significant changes of less than 0.5-fold 1 W and 2 W after ECLE (Figure 1). Table 1 shows the list of the top 30 genes that showed significant changes greater than 2.0-fold in all groups. The most highly upregulated gene common in the three samples was *Dcn*, which showed more than 100-fold higher expression than the control. Genes encoding collagen type V, I, III, and VI were included in the top 30 genes shown in Table 1. fibronectin 1 (*Fn1*), *Tpm2,* and *Tgfbi*, which are related to EMT, were also included in the top 30 genes shown in Table 1.

Gene Ontology analysis revealed that genes upregulated at both 1 W and 2 W were associated with defense response, response to stimuli, development of vasculature, extracellular matrix, regulation of cell migration, and response to stress (Table 2).

Table 3 lists the genes which were downregulated at 1 W and upregulated at 2 W after ECLE. Genes related to the differentiation of lens fiber, including gamma crystallin (Cryg) and filensin (Bfsp1), were initially downregulated and subsequently upregulated during PCO development.

Gene Ontology analysis revealed that genes downregulated at 1 W and upregulated at 2 W were associated with the structural constituent of the eye, lens development in the camera-type eye, lens fiber cell differentiation, and sensory perception (Table 4).

### 3.2. Confirmation of Gene Upregulation Using RT-qPCR and Western Blotting in Rat and Mouse PCO Models

**Figure 2:**
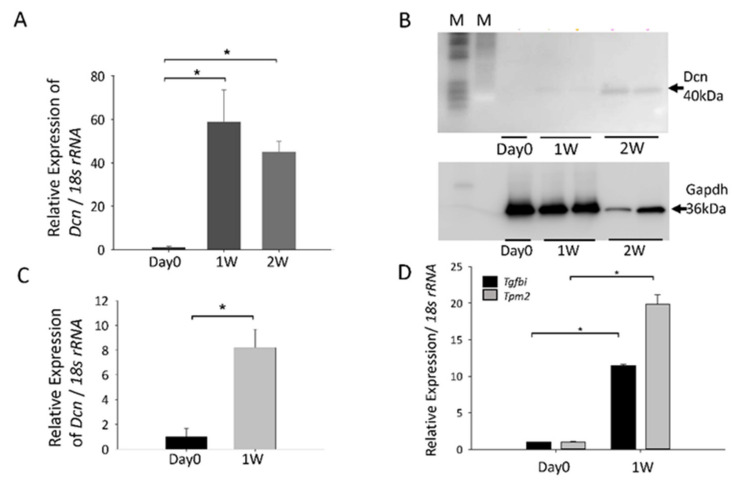
RT-qPCR validation of *Dcn* and *Tpm2* expression in rat or mouse posterior capsule opacification (PCO). (**A**) Expression of *Dcn* mRNA in rat PCO samples 1 and 2 weeks (W) after extracapsular clear lens extraction (ECLE) was compared to that on day 0 (* *p* < 0.0003) (**B**) Expression of *DCN* protein in mouse PCO samples 1 and 2 weeks after ECLE compared to that on day 0 (* *p* < 0.0025) (**C**) Expression of *Dcn* mRNA in mouse PCO samples 1 week after mouse PCO onset was compared to that on day 0 (* *p* < 0.0025) (**D**) Relative expression of *Tgfbi* and *Tpm2* mRNAs 1 week after mouse PCO onset was compared to that on day 0 (* *p* < 0.000001). Data were from three experiments and were reported as mean ± S.D (*n* = 3).

We confirmed the results of microarray using RT-qPCR and western blotting. Expression of the *Dcn* mRNA and DCN increased significantly in rat PCO at 1 and 2 W (Figure 2a,b; * *p* < 0.0003). Similarly, *Dcn* mRNA level increased significantly in mouse PCO at 1 W (Figure 2c; * *p* < 0.0025). Furthermore, the expression of *Tgfbi* and *Tpm2* mRNAs increased significantly in rat PCO 1 W after ECLE (Figure 2).

### 3.3. Effect of TGFβ and FGF2 on the Expression of Dcn and Tpm1 in MLECs and SRA-HLECs

TGFβ-2 and FGF2 are involved in the regulation of PCO and EMT. To investigate whether the expression of Dcn was affected in MLECs or SRA-HLECs treated with TGFβ or TGFβ plus FGF, we selected TGFβ-2, the major isoform expressed in eyes that contributes to PCO [1], and FGF2, the most potent of all FGFs, for further analyses.

**Figure 3:**
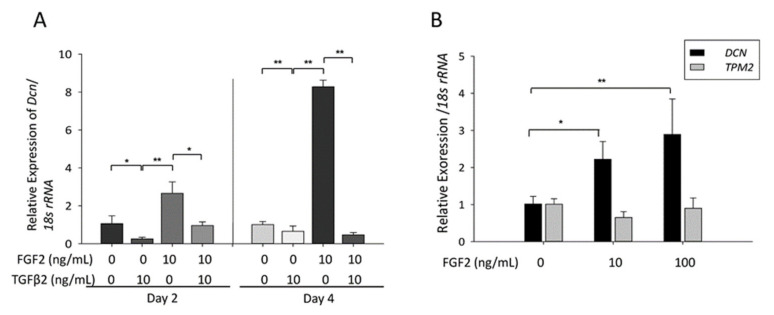
Expression of *Dcn* and *Tpm1* in cultured mouse lens epithelial cells (MLECs) and human mouse lens epithelial cells (HLECs) after treatment with FGF2 with/without TGFβ-2. (**A**) Cultured MLECs were plated in 35-mm dishes at the density of 1 × 10^5^ in Dulbecco’s modified Eagle’s media (DMEM) with 10% FBS for 24 h. LECs were treated with 10 ng/mL TGFβ-2 and/or 100 ng/mL FGF2 in DMEM containing 1% FBS for 2 and 4 days. The relative quantity of *Dcn* mRNA was determined using RT-qPCR analysis. * *p* < 0.004, ** *p* < 0.001. (**B**) Cultured HLECs were plated in 35-mm dishes at the density of 8 × 10^4^ in DMEM with 20% FBS for 24 h. LECs were treated with 0–100 ng/mL FGF2 in DMEM containing 1% FBS for 2 days. Relative quantity of *Dcn* and *Tpm1* mRNA was determined using RT-qPCR analysis. * *p* < 0.004, ** *p* < 0.05. Data were from three experiments and were reported as mean ± S.D. M: marker.

*Dcn* mRNA level increased in response to FGF2, and reduced in response to TGFβ-2 and a combination of FGF2 and TGFβ-2 on days 2 and 4 in MLECs (Figure 3a, * *p* < 0.05; ** *p* < 0.01). Furthermore, *Dcn* mRNA level increased in response to FGF2, and reduced in response to TGFβ-2 and a combination of FGF2 and TGFβ-2 on days 2 and 4 in MLECs (Figure 3a, * *p* < 0.05; ** *p* < 0.01). Similarly, the expression of *Dcn* mRNA increased significantly in SRA-HLEC after treatment with FGF2 (Figure 3b, * *p* < 0.004; ** *p* < 0.05).

### 3.4. Effect of hDCN Overexpression on TGFβ-Induced Tpm1 Upregulation

**Figure 4:**
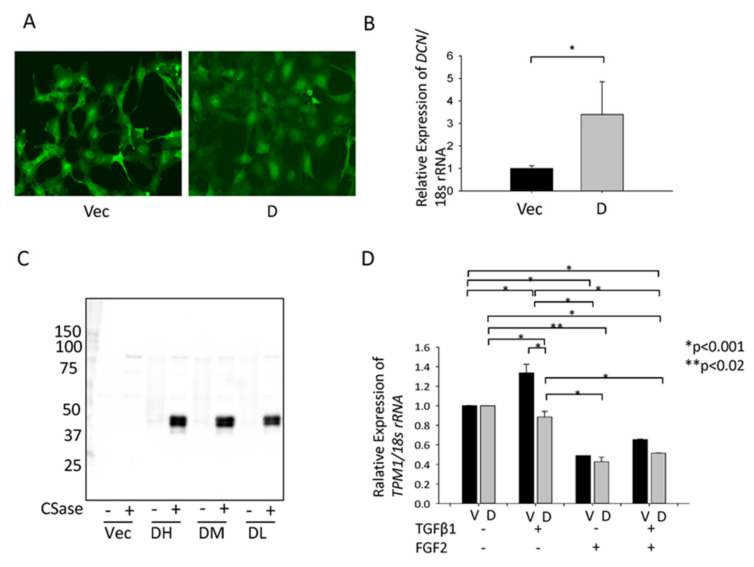
Effect of overexpression of hDCN in HLECs. (**A**) We monitored the transduction efficiency of GFP-vector (Vec) and GFP–hDCN (D) by observing the GFP-expressing cells within the live cell population. (**B**) RT-qPCR was performed to confirm the expression level of *hDCN* mRNA in GFP-vector (Vec) and GFP–hDCN (D)-overexpressing SRA-HLECs. * *p* < 0.05. (**C**) The collected culture supernatant was subjected to SDS-PAGE. In total, 250 ng protein was loaded per lane after concentrating the culture supernatant and digesting the GAG chain with or without protease-free chondroitinase (CSase). The secretion of DCN from SRA-HLEC overexpressing GFP–hDCN (Expression level: DH, High; DM, moderate; DL, Low) or GFP-Vec (Vec) was confirmed using western blotting. The core protein of DCN was approximately 50 kDa. (**D**) Cultured GFP-vector (V) or GFP–hDCN (D)-transfected SRA-HLECs were plated in 35-mm dishes at the density of 8 × 10^4^ in DMEM with 20% FBS for 24 h. LECs were treated with 10 ng/mL TGFβ-2 and/or 100 ng/mL FGF2 in DMEM containing 1% FBS and incubated for 2 days. TPM1 mRNA level was analyzed using RT-qPCR. PC; Positive control, human dermis (1 μg/lane). * *p* < 0.001, ** *p* < 0.02.

We observed that GFP was expressed in the GFP-vector and GFP-*hDCN*-transfected SRA-HLECs (Figure 4a). In GFP-hDCN-overexpressing SRA-HLECs, the expression of *hDCN* mRNA increased in SRA-HLECs and hDCN was secreted in the culture medium (Figure 4b,c; * *p* < 0.05). Expression of the *Tpm1* mRNA increased significantly in GFP-vector-transfected SRA-HLECs after treatment with TGFβ-2. However, *hDCN* overexpression significantly suppressed the upregulation of *Tpm1* mRNA in GFP-*hDCN*-transfected SRA-HLECs (Figure 4d). Alternatively, FGF2 treatment did not affect the expression of *Tpm1* in GFP-hDCN-overexpressing SRA-HLECs (Figure 4d; * *p* < 0.001, ** *p* < 0.02).

### 3.5. Lens Morphology of hDCN-Tg Mice

**Figure 5:**
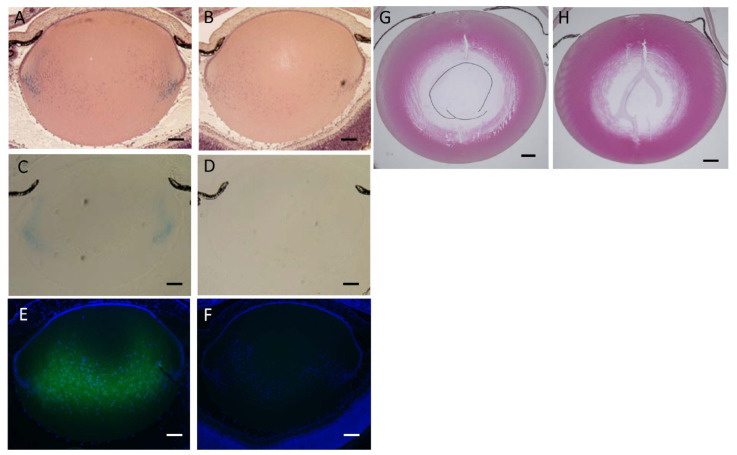
β-Galactosidase expression and histochemical analysis of lenses in *hDCN-Tg* animals. The findings regarding lens tissue in *hDCN-Tg* (**A**) newborn mice (PD1) did not differ from those in WT (**B**). (**C**) Lens images showing the expression of β-galactosidase in the anterior epithelial cells and primary lens fiber cells of the newborn *hDCN-Tg* mouse lens, revealed by X-gal staining. (**D**) Control sections from WT mice at PD1 were not stained with X-gal. (**E**) *DCN* was strongly immunostained in the lens fiber of the *hDCN-Tg* animal at PD1, suggesting that *DCN* secreted from LECs perfused into the lens fiber. (**F**) DCN immunostaining signal was undetectable or negative at PD1 in the lens of WT mice at PD1. Adult lenses in 25-week-old hDCN-Tg animals (**G**) did not show lens fiber damage and epithelial cell changes and did not differ from those in WT (**H**). Scale bar = 200 µm in a,b,c,d,e, and f; 100 µm in g and h.

Compared to that in WT mice, histological changes in the lenses on PD2 were not observed in *hDCN-Tg* mice (Figure 5a,b). Expression of *DCN* in *hDCN-Tg* animals on PD2 was confirmed using histochemical staining for lacZ with X-Gal and immunostaining using an anti-DCN antibody. As shown in Figure 5c, X-gal staining was observed in the cytoplasm of LECs at the equatorial to bow region and in the primary lens fiber in *hDCN-Tg* mice on PD. However, X-gal staining was not observed in the lens tissue of WT mice (Figure 5d). Similarly, DCN was strongly immunostained in newly formed lens fibers of *hDCN-Tg* animals on PD2 (Figure 5e). However, DCN was not immunostained in the lenses of WT mice (Figure 5f). The difference in the X-gal and anti-DCN immunostaining sites is because of the inability of X-gal to penetrate to the center of the lens. Histological changes were not observed in the lenses of 8-, 25-, and 48-week-old *hDCN-Tg* and WT mice (25-week-old mice: Figure 5g,h). These results suggested that hDCN-overexpression did not affect lens development and differentiation.

### 3.6. Effect of hDCN Overexpression on Wound Healing on Lens Surface In Vivo

**Figure 6:**
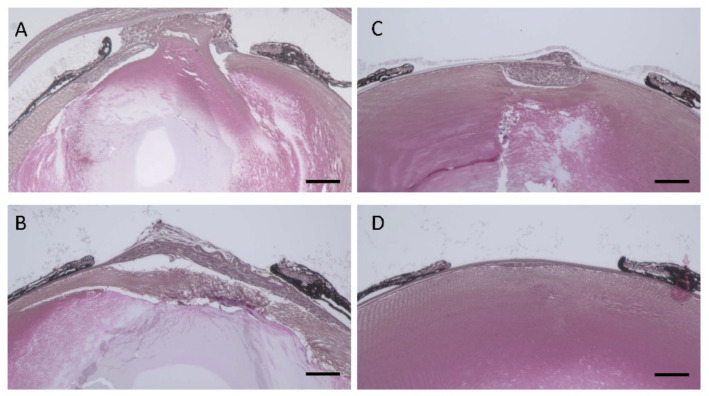
*hDCN*-*Tg* suppressed epithelial-mesenchymal transition (EMT) and fibroblastic changes in injured mouse lenses. Paraffin sections of the lenses of WT (**A**,**B**) and *hDCN*-*Tg* animals (**C**,**D**) with injury at 5 and 10 days were prepared and stained with H & E. Scale bar, 100 μm. Results are representative of six independent lenses.

The wounded areas in the lens surface revealed fibroblast-like tissue changes, indicating EMT in WT and *hDCN-Tg* animals (Figure 6). On days 5 (Figure 6a,c) and 10 (Figure 6b,d), fibroblastic changes around the wounded area were smaller in *hDCN-Tg* (Figure 6c,d) than in WT mice after needle puncture (Figure 6a,b).

**Figure 7:**
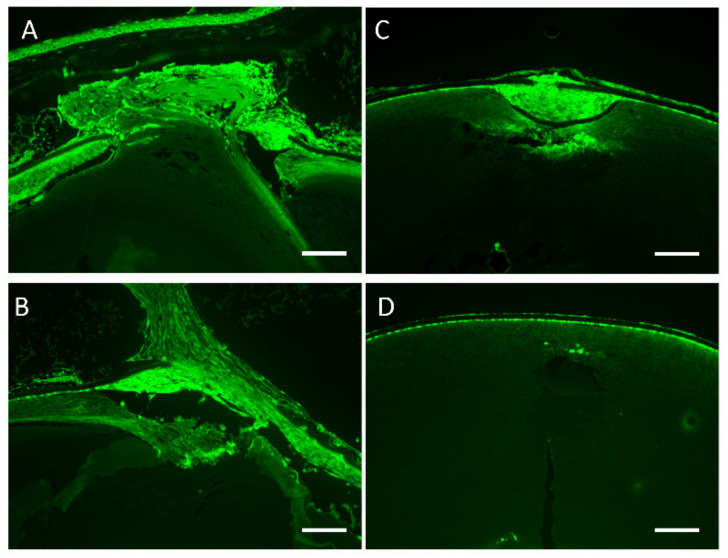
α-SMA was expressed in fibroblastic tissues observed in *hDCN-Tg* and WT animals. Paraffin sections of lenses of WT (**A**,**B**) and *hDCN-Tg* animals (**C**,**D**) with injury at 5 (**A**,**C**) and 10 (**B**,**D**) days were prepared and immunostained with anti-αSMA Ab. Scale bar, 100 μm. Results are representative of six independent lenses.

Fibroblastic LECs at the wound surface were immunopositive for αSMA on days 5 and 10 in both *hDCN-Tg* and WT mice (Figure 7a–d). The αSMA-immunopositive area in *hDCN-Tg* mice (Figure 7c,d) was smaller than that in WT mice (Figure 7a,b).

### 3.7. Relationship between Expression of DCN, Severity of Cataract, and Age in Aqueous Humor or Human LECs of Patients with Cataract

**Figure 8:**
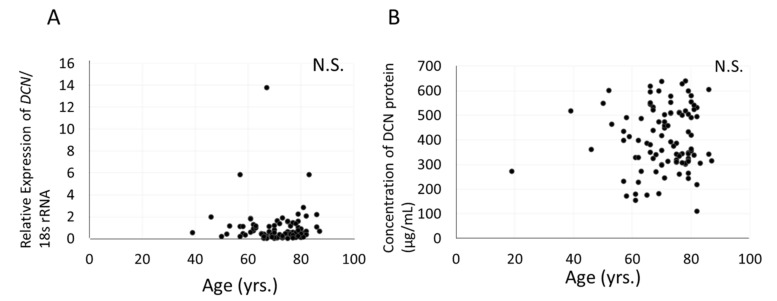
Relationship between age and *Dcn* mRNA expression in human LECs, and between age and concentration of DCN protein in human aqueous humor. RT-qPCR validation of *Dcn* mRNA expression in human LECs (**A**) and ELISA for DCN concentration in aqueous humor from patients with cataract (**B**) compared to that in the control (human LECs obtained from the clear lens after vitrectomy for removal of epiretinal membrane). N.S.: not significant; yrs: years-old.

We examined whether the expression levels of DCN were associated with human cataracts and aging. The sample size to enroll in this study was estimated by the adequate power analysis. However, no significant associations were noted between age (Figure 8a) or the severity of the three major types of cataracts (cortical, nuclear, and posterior subcapsular) and *Dcn* mRNA levels in human LECs of patients with cataract (data not shown). Although DCN was detected in human aqueous humor, its concentration varied among subjects, showing no association with age, cataract subtype, or grade (range of concentration: 111.0~639.2; mean: 403.5 ± 126.3). Significant associations between age (Figure 8b) or severity of the three major types of cataracts (cortical, nuclear, and posterior subcapsular) and DCN protein levels in human aqueous humor of patients with cataracts were not observed (data not shown).

## 4. Discussion

In the present study, we investigated the gene expression profiles of rat PCO to understand the mechanism underlying its progression. Using microarray analysis, we identified several LEC genes, expression of which was modulated in response to ECLE. Our experimental data showed that *Dcn* was highly upregulated in rat and mouse PCO. Furthermore, the expression of other PCO-related genes such as *Tgfbi*, a *Tgf-β*-induced extracellular matrix (ECM) [7,21], and *Tpm2*, also increased in DNA microarray analysis, which was confirmed using RT-qPCR in rat and mouse PCO. In our previous study, *Tpm1* and *Tpm2* were upregulated in the multilayered, spindle-shaped LECs in a rat model of PCO, human cataracts with anterior subcapsular fibrosis, and human differentiated LECs in a dislocated lens capsule [18]. In this study, *Tpm2* expression increased similarly in rat PCO, confirming that our rat and mouse PCO models were stable. Gene Ontology analysis revealed that the function of upregulated genes in rat PCO both 1 W and 2 W after ECLE were associated with defense response, response to stimuli, development of vasculature, extracellular matrix, regulation of cell migration, and stress response. After cataract surgery, genes related to the wound healing process appear to be induced rapidly in LECs. The functions of genes that were downregulated at 1 W and upregulated at 2 W after rat ECLE were associated with differentiation of lens fiber and lens development, such as γ-crystallin and filensin. After ECLE, EMT may start in the early stage of PCO and continue thereafter. In contrast, differentiation and regeneration of lens fiber may start late during rat PCO progression.

Using in situ hybridization, previous studies have shown that *DCN* mRNA is expressed in human postoperative LEC tissues with anterior capsular opacification [30]. In this study, *Dcn* was also highly expressed in rat and mouse PCO tissues. Initially, we speculated that DCN was related to EMT and fibrosis, similar to Tpm as reported previously [12,18,27]. Expression of Tpm increased after the addition of TGFβ-2 and decreased after the addition of FGF2 in cultured MLEC and SRA-HLEC (SRA01/04) [31]. However, our study revealed that the expression of *Dcn* mRNA increased after the addition of FGF2 and decreased after the addition of TGFβ-2 in cultured MLECs and SRA-HLECs. The effect of TGFβ-2 and FGF2 administration on Dcn expression was opposite to the effect on Tpm expression. DCN, a member of the small leucine-rich proteoglycan gene family containing leucine repeats with a glycosaminoglycan chain that harbors one chondroitin/dermatan sulfate (DS) side chain at its N-terminus, was originally named because of its ability to “decorate” collagen fibrils, thereby regulating fibrillogenesis, a key mechanism of matrix assembly and homeostasis [32,33]. Other studies have shown that DCN regulates the TGFβ signaling pathway and inhibits the growth of various tumor cells [34]. DCN suppresses tumor cell-mediated angiogenesis by inhibiting the endogenous production of vascular endothelial cell growth factor [35], similar to neutralizing antibodies directed toward the epidermal growth factor receptor (EGFR) [36]. Furthermore, DS from proteoglycans contributes to the signaling behavior of FGF2 [37] and hepatocyte growth factor/scatter factor [38]. Penc et al. (1998) demonstrated that DS released during wound repair can also activate FGF2 signaling [37]. FGF2 and TGFβ are related to the progression of PCO after cataract surgery [1,39,40]. After lens extraction surgery, FGF2 and TGFβ were upregulated in rabbit aqueous humor, while differentiation/proliferation and EMT of LEC were associated with PCO [40]. Hence, we speculated that DCN may be associated with the differentiation of LECs, but not EMT of LEC, as DCN expression increased in normal LEC treated with FGF2. We reported that TGFβ-2 induced epithelial-myofibroblastic transition in LEC and myofibroblast-like alternation with Tpm-positivity [12]. It is unclear whether DCN suppresses EMT, while DCN expression is reduced by TGFβ-2 in LEC, accompanied by EMT.

We generated *hDCN-Tg* mice to confirm the role of DCN in lens development, PCO, or EMT. Overexpression of *hDCN* in the lens of *hDCN-Tg* mice did not affect the development, growth, differentiation, and morphology of the lens. These results suggest that administration or overexpression of *DCN* did not adversely affect lens development and histology. In contrast, this study showed that pathological wound healing, such as fibroblastic EMT-like changes in a model of mouse lens injury was less severe in *hDCN-Tg* than in WT animals, suggesting that overexpression of *DCN* delays abnormal wound healing in mouse lenses and that DCN plays a protective role in the induction of pathological EMT in lenses. *DCN* expression increased in mouse and rat PCO tissues. We speculated that the upregulation of *DCN* was induced by FGF2, which may play a protective role against fibroblastic changes of LECs after cataract surgery. Reports show that DCN reduces subconjunctival tissue scarring [41] and intraocular pressure via fibrinolysis of the scarred trabecular meshwork [42] in animal models [43]. It modulates matrix metalloproteinase (MMP) activity by increasing levels of plasminogen [44], favoring higher ECM turnover and degradation. Indeed, DCN increases MMP-2 and MMP-9 levels, reduces extracellular matrix deposition [45], and attenuates fibrotic changes in animal models of many pathological conditions, including proliferative vitreoretinopathy [46], renal fibrosis [47], and spinal cord injury [48]. *DCN* is induced in residual lens epithelial cells after ECLE and participates in regulating PCO formation. The overexpression of *DCN* participates in regulating the anti-fibroblastic pathways to protect against surgical stress and participates in regulating the EMT, and the migration of residual capsular epithelial cells by inhibiting the action of TGFβ and Tpm. Thus, the addition or induction of DCN may inhibit the fibroblastic and EMT-associated changes observed in PCO.

Nevertheless, studies regarding *DCN* expression and the effects of its expression levels in human LECs before cataract surgery are lacking. DCN is present and secreted in human anterior aqueous humor and is expressed in human cataractous LECs. In age-related cataracts, DCN concentrations in human anterior aqueous humor and *DCN* expression in HLEC did not correlate with age, and opacity type or grade. It is of course cumbersome to analyze the level of *DCN* in human PCO samples and aqueous humor after cataract surgery in vivo. Nevertheless, the results of our in vitro and coupled with in vivo experimentation with *DCN* transgenic mice, adds weight that DCN is secreted into the aqueous humor and can play an important role in the human PCO or wound healing process in human LEC. We recognize that further studies are required to detail the exact contribution of DCN in human lens/LECs health.

Currently, there is no effective method for predicting who will develop PCO or to prevent its occurrence, and eradication of the disease remains an unmet goal in ophthalmology. Certain molecules, such as cytotoxic chemicals, or distilled water may non-specifically induce cell death during cataract surgery and influence the surrounding ocular tissues [49,50]. Aberrant TGF β signaling plays a major role in the EMT of cells akin to its role in the development of human PCO. Thus, drugs and molecules which inhibit TGF β signaling, myofibroblasts and EMT, should be considered for the treatment of PCO [27,31,51,52]. Based on the findings obtained in this study, overexpression of *DCN* in animal models could help further expand data that may ultimately lead to clinical studies and therapeutic interventions.

However, some limitations should be noted. First, experiments for the addition or injection of DCN are required, because DCN is a secreted protein. Thus, we are planning to produce a DCN recombinant protein for further study. Second, PCO *hDCN-Tg* mice should be generated and analyzed. Third, we have not analyzed the levels of human *DCN* expression by cataract grading, because the number of human samples was not adequate. It may be necessary to study the relationship between human cataracts and DCN.

## 5. Conclusions

In conclusion, this study revealed that the expression of *DCN* mRNA and protein increased in LECs in animal models of PCO. The development of new therapies for reducing fibrosis and pathological EMT induced by wound healing responses and PCO are necessary to maintain quality of vision after cataract surgery. We believe that administration of DCN or other surgical treatments will help in further reducing secondary cataract and surgical failure by minimizing postoperative PCO or scarring.

## Figures and Tables

**Table 1:** Lists of top 30 genes which were > 2.0 times up-regulated at 1 W and 2 W after ECLE.

	Gene Symbol	Gene Description		Gene Symbol	Gene Description
1	*Dcn*	decorin	16	*RT1-Da*	RT1 class II, locus Da
2	*Col5a2*	collagen, type V, alpha 2	17	*Cybb*	cytochrome b-245, beta polypeptide
3	*Fn1*	fibronectin 1	18	*Col6a3*	collagen, type VI, alpha 3
4	*Cyr61*	cysteine-rich, angiogenic inducer, 61	19	*Rnase4*	ribonuclease, RNase A family 4
5	*Col5a2*	collagen, type V, alpha 2	20	*Runx1*	runt-related transcription factor 1
6	*Col1a1*	collagen, type I, alpha 1	21	*Tpm2*	tropomyosin 2, beta
7	*Mylk*	myosin light chain kinase	22	*Ptgs2*	prostaglandin-endoperoxide synthase 2
8	*Col3a1*	collagen, type III, alpha 1	23	*Tgfbi*	transforming growth factor, beta-induced
9	*Actg2*	actin, gamma 2, smooth muscle, enteric	24	*Mgp*	matrix Gla protein
10	*Gbp2*	guanylate binding protein 2, interferon-inducible	25	*Ednrb*	endothelin receptor type B
11	*Emp1*	epithelial membrane protein 1	26	*Lyz2*	lysozyme 2
12	*Gpnmb*	glycoprotein (transmembrane) nmb	27	*Car3*	carbonic anhydrase 3
13	*Cd74*	Cd74 molecule, major histocompatibility complex	28	*Sfrp2*	secreted frizzled-related protein 2
14	*Dct*	dopachrome tautomerase	29	*Fcrls*	Fc receptor-like S, scavenger receptor
15	*Scn7a*	sodium channel, voltage-gated, type VII, alpha	30	*Ifitm1*	interferon-induced transmembrane protein 1

**Table 2:** Gene Ontology (GO) analysis; 1 W and 2 W > Day 0: > 2.0 times.

GO Accession Number	GO Term
GO:0006952|GO:0002217|GO:0042829	Defense response
GO:0009605	Response to external stimulus
GO:0051707|GO:0009613|GO:0042828	Response to other organisms
GO:0009607	Response to biotic stimulus
GO:0050896|GO:0051869	Response to stimulus
GO:0043207	Response to external biotic stimulus
GO:0001944	Vasculature development
GO:0031012	Extracellular matrix
GO:0030334	Regulation of cell migration
GO:0006950	Response to stress

**Table 3:** Lists of genes which were < 0.5 times down-regulated at 1 W and > 2.0 times up-regulated at 2 W after extracapsular clear lens extraction (ECLE).

Gene Symbol	Gene Description	1 W	2 W
*Crygb*	crystallin, gamma B	0.021	2.292
*Crygc*	crystallin, gamma C	0.139	4.448
*Crygd*	crystallin, gamma D	0.148	2.450
*Colq*	collagen-like tail subunit (single strand of homotrimer) of asymmetric acetylcholinesterase	0.300	2.324
*Bfsp1*	beaded filament structural protein 1, filensin	0.321	11.611
*Slc24a2*	solute carrier family 24 (sodium/potassium/calcium exchanger), member 2	0.323	5.878
*Snhg11*	small nucleolar RNA host gene 11 (non-protein coding)	0.380	6.667
*Slc46a3*	solute carrier family 46, member 3	0.423	2.121
*Anxa9*	annexin A9	0.466	2.296

**Table 4:** Gene Ontology (GO) analysis.

GO Accession	GO Term
GO:0005212	Structural constituent of eye
GO:0002088	Lens development in camera-type eye
GO:0007601	Visual perception
GO:0070306	Lens fiber cell differentiation
GO:0050953	Sensory perception of light stimulus
GO:0005198	Structural molecule activity
GO:0043010|GO:0001747|GO0031075	Camera-type eye development
GO:0001654|GO:0042460	Eye development
GO:0070307	Lens fiber cell development
GO:0007600	Sensory perception

## Data Availability

The data for the microarray analysis were deposited in the Gene Expression Omnibus (GEO) database (accession number: GSE152593).
